# Functional Lower Extremity Strength Influences Stepping Strategy in Community-Dwelling Older Adults During Single and Dual-Task Walking

**DOI:** 10.21203/rs.3.rs-3983607/v1

**Published:** 2024-03-15

**Authors:** Brandon M. Peoples, Kenneth D. Harrison, Keven G. Santamaria-Guzman, Silvia E. Campos-Varga, Patrick G. Monaghan, Jaimie A. Roper

**Affiliations:** School of Kinesiology, Auburn University, Auburn, AL, USA; School of Kinesiology, Auburn University, Auburn, AL, USA; School of Kinesiology, Auburn University, Auburn, AL, USA; University of Costa Rica, San Jose Province, San Pedro, Costa Rica; College of Pharmacy and Health Sciences, Wayne State University, Detroit, MI, USA; School of Kinesiology, Auburn University, Auburn, AL, USA

**Keywords:** mobility, lower extremity strength, gait, dual-task, stepping strategy

## Abstract

As age increases, a decline in lower extremity strength leads to reduced mobility and increased fall risks. This decline outpaces the age-related reduction in muscle mass, resulting in mobility limitations. Older adults with varying degrees of mobility-disability use different stepping strategies. However, the link between functional lower extremity strength and stepping strategy is unknown. Therefore, understanding how age-related reductions in functional lower extremity strength influence stepping strategy is vital to unraveling mobility limitations. Participants were recruited and tested at a local community event, where they were outfitted with IMUs and walked across a pressurized walkway. Our study reveals that older adults with normal strength prefer adjusting their step time during walking tasks, while those with reduced strength do not exhibit a preferred stepping strategy. This study provides valuable insights into the influence of functional lower extremity strength on stepping strategy in community-dwelling older adults during simple and complex walking tasks. These findings could aid in diagnosing gait deviations and developing appropriate treatment or management plans for mobility disability in older adults.

## Introduction

Maintaining mobility is vital for older adults to preserve their independence, self-reliance, community involvement, and overall health. Nevertheless, the aging process has a notable impact on this ability. As individuals cross their fifties, they may experience a gradual decline in their lower extremity strength, leading to reduced functional mobility, slower walking speeds, increased sedentary behavior, social isolation, higher fall risks, and a decline in their quality of life across physical, cognitive, emotional, and social domains ^1–3^. Alarmingly, lower extremity strength deteriorates faster than age-related reductions in muscle mass, prompting mobility limitations since walking requires supporting body weight through sit-to-stand transitions and propelling the body forward during walking using the lower body muscles ^3–5^. Therefore, mitigating deficits in lower extremity strength is paramount as society grays as it requires elucidating complex interactions between reduced lower extremity strength, destabilized gait, and heightened mobility-disability.

Functional lower extremity strength plays a critical role in preserving mobility but inevitably declines with advanced age, resulting in a nearly three-fold increase in fall risks in older adults and costing over $50 billion in annual geriatric injury expenditures from government programs such as Medicare ^6,7^. Quantifying decrements to functional lower extremity strength before outright mobility-disability occurs is critical but requires direct assessments linking functional lower extremity strength to gait safety. The 5-repetition Sit-to-Stand test fills this need since itis a clinically feasible test of functional lower extremity strength that holds validity and strongly predicts mobility limitations in older adults ^8–11^. Poor performance on the 5xSTS is associated with reduced gait speed, functional impairment, frailty, and fall risk in community-dwelling adults ^1,8,11–14^. Moreover, older adults with reduced functional mobility use different stepping strategies (increasing step length or step time); however, the link between functional lower extremity strength and stepping strategy is unknown ^15^. Therefore, understanding how age-related reductions in functional lower extremity strength influence stepping strategy is vital to unraveling mobility limitations.

Stepping strategy is important to contemplate while examining normal and dual-task walking patterns. In order to gain a comprehensive understanding of how older adults navigate their environment, we need to consider two important factors: the ability to regulate walking pace and the aptitude to perform multiple tasks simultaneously while walking ^16,17^. Regulating walking pace is important to meet the demands of the environment. However, the ability to walk and multitask is essential for maintaining independence, as many daily activities require the ability to walk while performing other tasks, such as carrying a plate of food or engaging in conversation ^16,18–20^. Thus, examining the relationship between functional lower extremity and stepping strategy in older adults during simple or complex walking tasks can provide valuable insights into gait safety.

This study investigates whether functional lower extremity strength affects stepping strategy in community-dwelling older adults across normal and dual-task walking. We hypothesize: 1) Older adults with reduced functional lower extremity strength will use a step time dominant stepping strategy than those with normal strength, and 2) Stepping strategies will be similar between groups during walking conditions where multitasking is required. Thus, investigating functional lower extremity strength and stepping strategy can enhance our understanding of the factors that impact gait stability and help devise effective interventions to proactively address mobility-disability.

## Methods

### Participants

Our study’s participant recruitment and data collection were conducted at a local community health fair event instead of a laboratory. This was done to improve ecological validity and demonstrate the feasibility of implementing assessment procedures in a practical, real-world context. The recruitment and data collection took place over two consecutive years (2022 and 2023) as part of our ongoing research engagement and service partnership to address health needs in a rural region. Our study focused on a sample from an underserved rural community. It was in response to a call from the National Institute of Aging (NIA) workshop on age-related changes in gait biomechanics. The workshop stressed the importance of diverse sampling and building community partnerships ^21^. Twenty older adults (17F, 72 ± 6) living in the Lee County community participated in this study during a local health fair. Participants who were free from lower extremity injuries such as bone fractures, muscle strains, and joint dislocations were included in the study. Participants who did not report any severe problems that may affect their ability to walk were also included. However, those who reported any neurological disease such as Parkinson’s disease, Essential Tremor, Multiple Sclerosis, Stroke, or Traumatic Brain injury were excluded from the study. Almost 50% of the participants in the study were from communities of color.

### Material

This study was approved by the Auburn University Institutional Review Board on the basis of minimal risk, and a waiver for informed consent was granted. All participants were given a detailed explanation of the study’s purpose, procedures, potential risks, and benefits, and provided informed consent before their inclusion in the study. The study was conducted according to the ethical principles outlined in the Declaration of Helsinki. Following consent participants underwent a series of assessments, including a set of questionnaires, two strength assessments, three functional mobility assessments, and four gait assessments. The questionnaire included questions about their demographic information, living status, education, sleep habits, retrospective fall history, and self-reported physical activity history. Fear of falling was measured using the Fall Efficacy Scale (FES) developed by Tinetti et al. (1990), and barriers to physical activity were assessed using the CDC Barrier to Being Active Quiz (BBAQ) (Control & Prevention, 2013).

### Functional Lower Extremity Strength measured by the Instrumented 5-Rep Sit-to-Stand (i5xSTS)

The i5xSTS device was employed to measure the functional strength of the lower extremities using wireless inertial measurement units (IMU) from APDM Opals (APDM Inc, Portland, OR). The Opal sensor has triaxial accelerometers, gyroscopes, and magnetometers, capturing signal data at 128 Hz. To obtain readings, researchers placed six IMUs at four different body locations: 1) atop the sternum centered over the manubrium, 2) on the superior aspect of the posterior sacral surface (below the fifth lumbar vertebrae L5), 3) on the posterior aspect of the distal radius and ulna, and 4) on the dorsal surface of the foot, centered on the intermediate and lateral cuneiform. Participants were instructed to sit with their backs against the chair, arms crossed, and hands touching the anterior aspect of their deltoid. They were then asked to stand and sit as quickly as possible for five repetitions. The duration of this exercise was used to determine the strength status of the participants in this study.

### Gait Testing

All participants completed the walking trials on a walkway next to the main health fair venues and were exposed to roughly ~ 75dB of sound. All assessments were conducted during vendor exhibitions, demonstrations, and community events to reflect real-world complexity. Participants were asked to walk under four conditions during the community health fair. These were: 1)Walking at their normal speed, 2) Walking at their fastest speed, 3) Motoric Dual Task at their normal speed (Walking at their normal speed while holding a tray with a cup of water), and 4) Motoric Dual Task at their fastest feed (Walking at their fastest speed while holding a tray with a cup of water. The researcher instructed the participants to walk at either a “comfortable, natural walking speed” or the “fastest, safe walking speed without jogging or running,” as prompted. Additionally, the researchers provided non-prioritizing instructions while the participants walked with the tray to prevent participants from prioritizing the tray task over the walking task, and vice versa. The GAITRite instrumented walkway was used to capture all spatial and temporal gait cycle parameters used for this study. Our GAITRite system (GAITRite Gold, CIR Systems, Clifton, NJ) consists of an electronic walkway approximately 8.2 meters long, connected to a personal computer via an interface cable. The walkway comprises a series of sensor pads inserted in a grid formation between a layer of vinyl (top cover) and foam rubber (bottom cover). The active area of the walkway is 61 cm wide and 732 cm long, while the sensors are placed 1.27 cm apart, consisting of a total of 27,648 sensors that are activated by mechanical pressure. The data from the activated sensors is collected by a series of onboard processors and transferred to the computer through a serial port. The system has a sampling rate of 80 Hz.

## Data analysis

### Functional Lower Extremity Strength measured by the Instrumented 5-Rep Sit-to-Stand (i5xSTS)

i5xSTS was collected from APDM Opals and participant data was processed in Moveo Explorer version 1.0.0.202206 (APDM Inc, Portland, OR). Total duration was calculated using the average duration of the sit-to-stand and stand-to-sit transitions. Participants were classified as low strength (LS) if their 5xSTS duration exceeded normative performance values for their age range of 11.4 seconds (60 to 69 years), 12.6 seconds (70 to 79 years), and 14.8 seconds (80 to 89 years) ^22^.

### Gait Cycle Parameters

Gait speed, step length, and step time for all walking conditions were exported from the GAITRite software (GAITRite, CIR Systems Inc., Clifton, NJ). Step length was measured using the heel center of the current footprint to the heel center of the previous footprint on the opposite foot. Step time is the elapsed time from the first contact of one foot to the first contact of the opposite foot. Gait Speed was measured using the distance traveled divided by the total ambulation time. Step length and step time from each condition was used to calculate the stepping strategy measured by Length-Time Difference (see below).

### Length-Time Difference (LTD)

LTD was derived from a previous study evaluating how strategy, adjustments to cadence or stride length when walking at a normal speed and fast speed, influence lower extremity joint moments ^23^. The equation used to calculate strategy for cadence and stride length are below:

Ardestani’s Strategy Eq. (1)

$$\Delta {\mathrm{Cadence}}_{i}=\left(\frac{{\;\mathrm{Cadence}}_{\mathrm{Fast}}-{\mathrm{Cadence}}_{\mathrm{Normal}}}{{\mathrm{Cadence}}_{\mathrm{Normal}}}\right)*100\%i=\mathrm{numberofsubjects}\;\;\Delta {\mathrm{Stride}}_{i}=\left(\frac{{\mathrm{Stride}}_{\mathrm{Fast}}-{\mathrm{Stride}}_{\mathrm{Normal}}}{{\mathrm{Stride}}_{\mathrm{Normal}}}\right)*100\%$$

**Figure 1:**
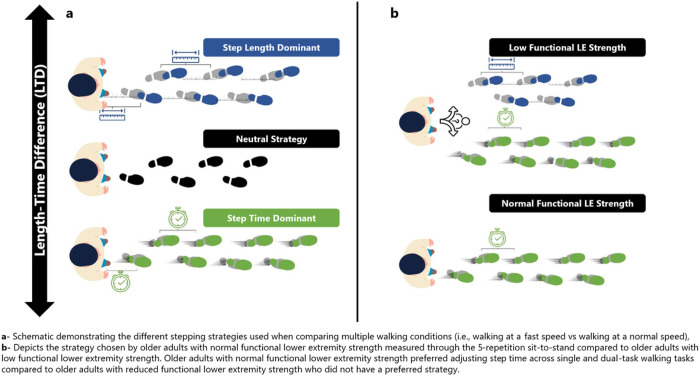
Schematic demonstrating the different stepping strategies used when comparing multiple walking conditions.

LTD represents relative change in step length and step time when comparing two conditions (i.e. fast vs preferred. An LTD > 0 indicates a step length dominant strategy (increasing step length), an LTD < 0 indicates a step time dominant strategy, while an LTD = 0 indicates a neutral strategy, implying equal contribution from both step time and step length (see Fig. 1) ^15,23^.

Length-Time Difference Eq. (2)

$$LTD=\left(\frac{{\mathrm{Length}}_{\mathrm{Fast}}-{\mathrm{Length}}_{\mathrm{preferred}}}{{\mathrm{length}}_{\mathrm{preferred}}}\right)+\left(\frac{{\mathrm{Time}}_{\mathrm{Fast}}-{\mathrm{Time}}_{\mathrm{preferred}}}{{\mathrm{Time}}_{\mathrm{preferred}}}\right)*100\%$$

Modified Length-Time Difference Eq. (3)

$$LTD=\left(\frac{{\mathrm{Length}}_{\mathrm{Tray}+\mathrm{WalkSpeed}}-{\mathrm{Length}}_{\mathrm{WalkSpeed}}}{{\mathrm{length}}_{\mathrm{WalkSpeed}}}\right)+\left(\frac{{\mathrm{Time}}_{\mathrm{Tray}+\mathrm{WalkSpeed}}-{\mathrm{Time}}_{\mathrm{WalkSpeed}}}{{\mathrm{Time}}_{\mathrm{WalkSpeed}}}\right)*100\%$$

Following the equations above, we calculated the stepping strategy for four distinct comparisons: Walking at a Fast Speed vs Walking at a Normal Speed (Walking Comparison 1), Motoric Dual Task at a Fast Walking Speed vs Motoric Dual Task at a Normal Walking Speed (Walking Comparison 2), Motoric Dual Task at a Normal Walking Speed vs Walking at a Normal Speed (Walking Comparison 3), and Motoric Dual Task at a Fast Walking Speed vs Walking at a Fast Speed (Walking Comparison 4).

### Statistical Analysis

#### Power Analysis

An *a priori* power analysis was conducted using G*Power version 3.1.9.7 ^24^ to attain 80% power and determine the minimum sample size based on data from ^15^, which observed nearly large effect size (*d* = 0.77) between the 5-repetition chair stand duration of the Short Physical Performance Battery and stepping strategy using Length-Time difference in older adults with mobility limitations. With the significance criterion set at *α* = .05 and power = .80, the minimum sample size needed with this effect size is *N* = 12 for a repeated measures ANOVA.

##### Descriptive Statistics

Descriptive statistics are provided for each group for the following variables: gait speed (m/s), step length (m), and step time (s).

##### Independent Samples T-Test

An independent samples t-test was conducted to compare age, height, mass, 5x sit-to-stand test duration, sit-to-stand duration, stand-to-sit duration, sit-to-stand angle, stand-to-sit angle, and Falls Efficacy Scale (FES) score between the low strength and normal strength groups.

##### 2×4 Repeated Measures ANOVA

A 2×4 repeated measures ANOVA was conducted to examine the effect of group (Low vs. Normal) and walking comparisons: Walking at a Fast Speed vs Walking at a Normal Speed (Walking Comparison 1), Motoric Dual Task at a Fast Walking Speed vs Motoric Dual Task at a Normal Walking Speed (Walking Comparison 2), Motoric Dual Task at a Normal Walking Speed vs Walking at a Normal Speed (Walking Comparison 3), and Motoric Dual Task at a Fast Walking Speed vs Walking at a Fast Speed (Walking Comparison 4) measured by LTD. Mauchly’s test indicated that the assumption of sphericity was violated for condition (p < .05); therefore, we used Greenhouse-Geisser correction. Post-hoc test comparisons were performed using the Tukey HSD test.

## Results

### Descriptive Statistics

**Figure 2:**
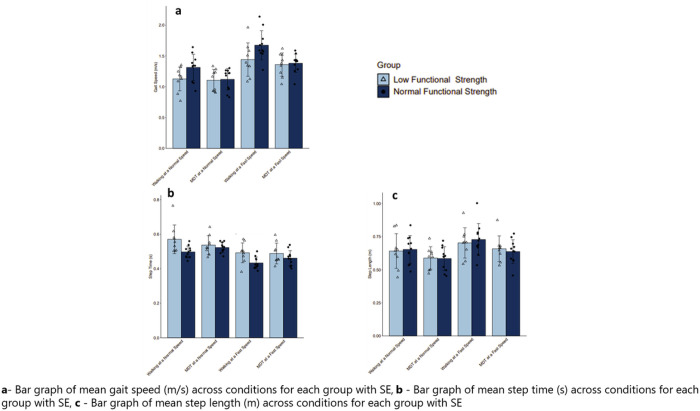
Box plots of stepping strategy across walking comparison.

The low functional strength group had a slower gait speed (*M* = 1.26, *SD* .25) across the single and motoric dual task walking trials compared to the normal strength group (*M* = 1.37, *SD* .28). The step lengths between each group were similar (*M* = .65, *SD* .11), however, the normal functional strength group had longer overall stepping times (*M* = .48, *SD* .05) in comparison to the low functional strength group (*M* = .38, *SD* .77), summarized in Table 1. Figure 2 depicts mean gait speed (m/s), step length (m), and step for each group across single and motoric dual task walking conditions.

### Independent Samples T-Tests

An independent samples T-test was conducted to compare group characteristics summarized in Table 1. There was a significant difference in 5x sit-to-stand test duration between the low strength group (*M* = 19.35, *SD* = 4.73) and the normal strength group (*M* = 11.27, *SD* = 2.21), *t*(18) = 5.05, *p* < .001, *d* = 2.27. This indicates that the low-strength group took significantly longer to complete the 5x sit-to-stand test than the normal-strength group with an extremely large effect size. Longer 5x sit-to-stand test duration may be attributed to the long sit-stand phases (*p* < .006) and greater sit-to-stand lean angles (*p* < .006). No significant differences existed between age, height, mass, Stand-to-sit duration, Stand-to-Sit lean angle, or FES score, summarized in Table 2.

### 2 × 4 Repeated Measures ANOVA

**Figure 3:**
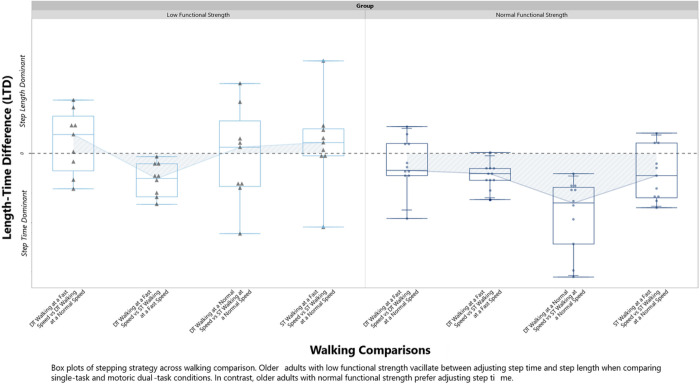
Paneled Bar graphs of gait speed, step time, and step length for each group across walking conditions.

A 2 × 4 repeated measures ANOVA showed statistically significant differences in the main effects of stepping strategy, functional lower extremity strength (Low vs. Normal) and interaction between functional lower extremity strength and stepping strategy measured by LTD, summarized in Table 3. The normal strength group showed a clear preference for a step time dominant strategy (*M* = −7.17, *SD* = 3.82) compared to the low strength group. The low strength group vacillated between step length and step time across walking comparisons (*M* = −0.4, *SD* = 4.94) see Fig. 3.

### Figure

Post hoc analysis with a Tukey’s HSD revealed there was a mean difference of 14.32 (95% CI: 0.68 to 3.60) between the Normal Strength Walking and Low Strength groups during Walking Comparison 3 (p < .001). Tests of simple main effects showed the interaction was driven by differences between strength groups in Walking Comparison 3 (Motoric Dual Task at a Normal Walking Speed vs Walking at a Normal Speed), *F*(1, 18) = 9.66, p < .05, summarized in Table 4.

## Discussion

The present study investigated if functional lower extremity strength influences stepping strategy in community-dwelling older adults during simple and complex walking. Our main findings are: 1) Older adults with normal lower extremity strength preferred adjusting their step time when walking in both single and dual task conditions, 2) Older adults with reduced functional strength did not exhibit a preferred stepping strategy while walking, either with or without a motoric dual task (i.e., walking while holding a tray balancing a cup of water), 3) The interaction of stepping strategy was mostly influenced by the motoric dual task.

Our findings demonstrate that adults with reduced functional lower strength lack a preferred stepping strategy across all walking comparisons, whereas stronger older adults prioritize adjusting step timing over step length. Adjusting step timing may be a safer compensation for older adults to regulate walking given age-related declines in neuromuscular control, balance, proprioception, and reaction time ^25,26^. Relying on adjusting step length likely reduces instability by bringing the center of mass closer to the leading foot to improve stability ^27^.

Contrary to our hypothesis, the low strength group did not demonstrate a step time strategy. Upon examination of the individual data, over half of the people in the low strength group utilized a step length strategy. Our findings disagree with Baudendistel et al. (2021) who reported in a sample of people with mobility disability, a longer sit to stand time relates to more step timing adjustments when comparing a walking trial at a comfortable pace versus a walking trial at a faster pace. Our differences between studies can be explained by the walking conditions used, and population sampled. Specifically, Baudendistel et al. recruited people who met requirements for mobility disability, as determined by physical inactivity, worse physical function, and slower preferred gait speed. Our study did not have the same exclusion and inclusion criteria, and we recruited people at a local health fair hosted in a community recreation center, and 13 of 20 participants reported being physically active ^28^. Additionally, Baudendistel et al. compared a single task preferred-walking speed to a single task fast-walking speed. Our study adds complexity to this comparison by having participants complete a motoric dual task during fast and normal walking, in addition to single task fast and normal walking. Interestingly, the interaction effect in our study reveals functional lower extremity strength impacts stepping strategy primarily when attention is divided during walking - an essential skill for navigation and avoidance of fall risk hazards ^29^.

Elucidating connections between functional lower extremity strength, attentional resources, and stepping strategies provides clinically meaningful patient profiles of gait instability often overlooked in older adults without overt mobility disability. Tailoring gait retraining programs by strength capacity and attention underscores a precision rehabilitation approach to improve neurocognitive control of gait and mitigate fall risk ^30^. Future research should explore how strength training combined with stepping strategy re-education impacts gait variability and dual-task.

A strength of our study lies in its community-based approach, which fosters inclusivity compared to traditional university settings. By engaging individuals in community settings away from the university, we enhance the generalizability of our findings. This approach addresses limitations associated with convenience samples from local university communities^21^. Actively seeking participation from the community ensures our research reflects a broader spectrum of experiences, enriching the validity and applicability of our results. Our community-based approach fosters collaboration, enhances diversity, and ensures interventions meet specific needs, aligning with principles of community engagement.

## Conclusion

In our study, we found that functional lower extremity strength assessed by the instrument 5-repetition Sit-to-Stand influences stepping strategy in community-dwelling older adults. Older adults with normal functional lower extremity strength utilize a step-time dominant strategy, adjusting step time under normal and dual-task conditions. In contrast, older adults with reduced lower extremity strength lack a clear preference for modulating step length or time to walk faster. When faced with an added cognitive demand during gait, older adults with reduced lower extremity strength switch towards a step-length dominant strategy, compared to older adults with normal strength who maintain a time-based strategy. Our findings can help clinicians proactively identify community-dwelling older adults with reduced lower extremity strength who may have difficulty safely adjusting gait patterns in response to environmental demands. Future research should test whether strength training programs can also optimize stepping strategies during walking in older adults with reduced lower extremity strength.

## Figures and Tables

**Table 1:** Means and Standard Deviations for Gait Speed (m/s), Step Time (s), and Step Length (m) for each group across all walking conditions.

	Low Strength		Normal Strength	
Gait Speed (m/s)	1.26 ± .25	.77–1.96	1.37 ± .28	0.831–2.14
Step Length (m)	0.65 ± .11	.44 – .93	.65 ± .11	.45–1.0
Step Time (s)	0.38 ± 0.77	1.45–1.73	.48 ± .05	.39 – .56

**Table 2:** All Participant Characteristics

	Low Strength		Normal Strength		*p*	Cohen’s *d*
Sex (M/F)	2M/7F		1M/10F		.46	0.36
Age (years)	74 ± 7	65–87	69 ± 5	61–78	.07	0.87
Height (m)	1.64 ± 0.1	1.45–1.73	1.61 ± .1	1.47–1.79	.29	0.29
Mass (kg)	84.1 ± 15.5	63.4–113.8	74.7 ± 14.6	52.6–101.3	.24	0.62
5xSTS Duration (s)	19.3 ± 4.7	15.3–27.6	11.3 ± 2.2	7.7–14.1	**< .001**	2.27
Sit-to Stand duration	1.4 ± 0.6	0.9–2.8	0.9 ± 0.17	0.7–1.2	**.006**	1.39
Stand-to-Sit duration	0.90 ± 0.4	0.6–1.8	0.75 ± 0.3	0.6–1.5	.32	0.46
Sit-to-Stand lean angle	57 ± 28	21–104	29 ± 6	21–38	**.006**	1.41
Stand-to-Sit lean angle	32 ± 10	14–101	28 ± 6	23–28	.08	0.83
FES Score	17 ± 8	10–29	14 ± 6	10–30	.36	0.46

*p*-values in bold indicate statistically significant differences between groups. Values indicate mean ± sd. Values indicate range: minimum-maximum. M, male, F, female. FES-Falls Efficacy Scale

**Table 3:** Main Effects of Repeated Measures ANOVA

Factor	*F* (df)	*p*	*η^2^_p_*
Walking Comparison	5.205 (2,36)	**0.010**	0.224
Strength Status	11.773 (1, 18)	**0.003**	0.395
Walking Comparison * Strength Status	3.487 (2,36)	**0.041**	0.162

**Table 4:** Simple Main Effects - Strength Status

Level of Condition	Sum of Squares	df	Mean Square	F	p
Walking Comparison 1	310.211	1	310.211	4.057	0.059
Walking Comparison 2	147.660	1	147.660	2.933	0.104
Walking Comparison 3	1025.323	1	1025.323	9.661	**0.006**
Walking Comparison 4	4.166	1	4.166	0.308	0.586

## Data Availability

The datasets generated during and/or analyzed during the current study are available from the corresponding author upon reasonable request.
